# A New Predictive Model of Centerline Segregation in Continuous Cast Steel Slabs by Using Multivariate Adaptive Regression Splines Approach

**DOI:** 10.3390/ma8063562

**Published:** 2015-06-17

**Authors:** Paulino José García Nieto, Victor Manuel González Suárez, Juan Carlos Álvarez Antón, Ricardo Mayo Bayón, José Ángel Sirgo Blanco, Ana María Díaz Fernández

**Affiliations:** 1 Department of Mathematics, University of Oviedo, Oviedo 33007, Spain; 2 Department of Electrical Engineering, University of Oviedo, Gijón 33204, Spain; E-Mails: vmsuarez@uniovi.es (V.M.G.S.); anton@uniovi.es (J.C.A.A.); rmayo@uniovi.es (R.M.B.); sirgo@uniovi.es (J.A.S.B.); 3 Finishing Department, ArcelorMittal España, Avilés 33400, Spain; E-Mail: ana.diaz@arcelormittal.com

**Keywords:** statistical learning techniques, continuous cast steel labs, centerline segregation, multivariate adaptive regression splines (MARS), regression analysis

## Abstract

The aim of this study was to obtain a predictive model able to perform an early detection of central segregation severity in continuous cast steel slabs. Segregation in steel cast products is an internal defect that can be very harmful when slabs are rolled in heavy plate mills. In this research work, the central segregation was studied with success using the data mining methodology based on multivariate adaptive regression splines (MARS) technique. For this purpose, the most important physical-chemical parameters are considered. The results of the present study are two-fold. In the first place, the significance of each physical-chemical variable on the segregation is presented through the model. Second, a model for forecasting segregation is obtained. Regression with optimal hyperparameters was performed and coefficients of determination equal to 0.93 for *continuity factor* estimation and 0.95 for *average width* were obtained when the MARS technique was applied to the experimental dataset, respectively. The agreement between experimental data and the model confirmed the good performance of the latter.

## 1. Introduction

It is well-known that the segregation is a phenomenon appearing during the solidification of metals and alloys which consists in a non-uniformity of the chemical composition due to the fact that the material contains solutes, which are more soluble in the liquid than in the solid, so, when the solidifying front advances, those solutes are rejected from the solid and enrich the liquid [1,2,3,4,5,6,7].

The continuous casting of slabs is aimed at producing a product with a proper chemical composition, geometry and surface quality, without any or a minimum acceptable level of external and internal defects. One of the most unpredictable defects of the slabs is centerline segregation, which has a negative effect on further processing of the slabs and hence on the possible uses of the final product [7,8,9,10,11,12,13].

All metal castings experience segregation to some extent and segregation can be classified into micro-segregation and macro-segregation. Micro-segregation takes place at the level of the microstructure of the material and it refers to localized differences in composition between dendrite arms, and can be significantly reduced by a homogenizing heat treatment. This is possible because the distances involved (typically on the order of 10 to 100 µm) are sufficiently small for diffusion to be a significant mechanism. This is not the case in macro-segregation. The macro-segregation takes place due to the movement of the micro segregated regions on macroscopic distances due to the movement of the liquid and free crystals. Therefore, macro-segregation in metal castings cannot be remedied or removed using heat treatment. Specifically, this research work studies one type of macro-segregation, the central segregation, in a continuous cast steel slabs. It appears as a line of impurities in the central line of a transversal section of the slab. In this central area cracks could also appear, which can be very harmful when slabs are rolled to thick plate [1,2,3,4,5,6,7,8,9,10,11,12,13].

The aim of this research is to construct a multivariate adaptive regression splines (MARS) model to identify central segregation in continuous cast steel slabs. Multivariate adaptive regression splines (MARS) technique is a form of regression analysis introduced by Jerome Friedman in 1991 [14,15,16,17,18,19,20,21,22,23]. It is a non-parametric regression technique and can be seen as an extension of linear models that automatically models nonlinearities and interactions, as those analyzed successfully in this innovative research work. According to previous research, the MARS technique has been proven to be an effective tool to predict natural parameters, being successfully used in a wide range of fields, such as forest modeling [20], estimation of the battery state-of-charge [21], prediction of the building energy performance [22], assessment of soil liquefaction [23], and so on.

The purpose of this work may be classified as a problem of modeling/forecasting where the value of a target variable is predicted from input data or process variables. Specifically, in this study we are going to predict the defect of the central macro-segregation in steel slabs.

Steel is an alloy of iron and carbon that is widely used in construction and other applications because of its hardness and tensile strength. Carbon, other elements, and inclusions within iron act as hardening agents that prevent the movement of dislocations that naturally exist in the iron atom crystal lattices. The carbon in typical steel alloys may contribute between 0.03% and 1.075% of its weight and up to 2.1% maximum. Alloys with a higher than 2.1% carbon content, depending on other element content and possibly on processing, are known as *foundries* or *cast iron*. Cast iron is not malleable even when hot, but it can be formed by casting, as it has a lower melting point than steel and good castability properties. Therefore, the main difference between the iron and steel is the percentage of carbon: steel is iron with a carbon percentage between 0.03% and 1.075%. Above this percentage, iron alloys are considered [1,2,3,4,5,6,7,8,9,10,11,12,13].

Impurities are all undesirable additional elements into the composition of steels. They are found in steels and also in cast irons because they are present in the mineral (raw material) and fuels. It is very important to eliminate or reduce their content because they are detrimental to the properties of the alloy. When their elimination is not possible or it is too expensive, their presence is allowed in minimal amounts.

Segregation is a phenomenon that depends on the steel composition (solutes) and the cooling conditions of the steel slab. Therefore, the input variables of the model are mainly related to these two parameters. The variables related to primary and secondary steelmaking are discarded since they have no influence on the steel solidification [1,2,3,4,5,6,7,8,9,10,11,12,13].

This innovative research work is organized as follows. Firstly, the necessary materials and methods to carry out this study are described. Secondly, the obtained results are shown and discussed. Finally, the main conclusions drawn from the results are exposed.

## 2. Materials and Methods

### 2.1. Experimental Dataset

**Figure 1:**
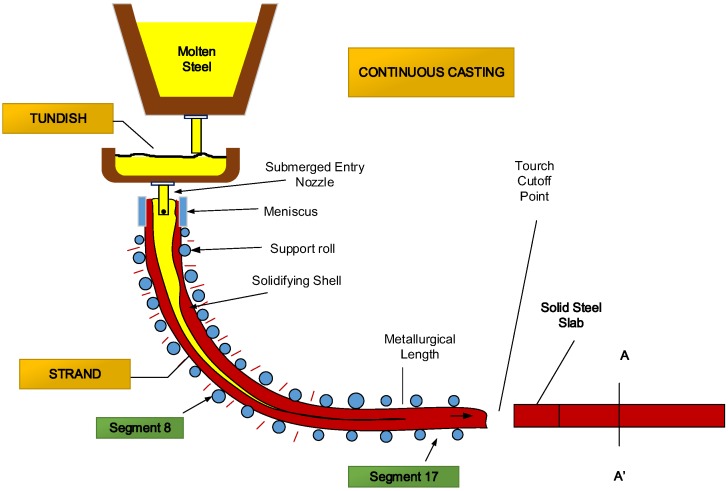
Schematic diagram of the continuous casting of steel slabs.

The dataset used for the MARS analyses was collected using a database from the continuous casting process of steelmaking belonging to the company Arcelor-Mittal located in Avilés (Northern Spain). This database contains the variables related to the process of solidification of steel slabs (see Figure 1).

The main goal of this research work was to obtain the dependence relationship of segregation factor (output variable), as a function of the input variables. As said before, hundreds of variables are involved in a process such as the continuous casting of steel. A first task in the model development is the selection of input and output variables. Output variables are two indexes given by the tool used to evaluate segregation from sulfur prints: *Continuity factor* (C factor) and *Average width*. C factor is a measure of the continuity of the segregated band and Average width is the average width of the spots forming the centerline segregation [1,2,3,4,5,6,7,8,9,10,11,12,13].

Input variables have been selected among all the ones controlled in the casting process. They include: steel composition in the tundish, temperature and superheating of the steel, casting speed, mold cooling, secondary cooling, *etc*. It is known from the experience that some factor have a bigger influence on segregation than others, so that the variable selection was done based on this previous knowledge of the process resulting in the following group of input variables [1,2,3,4,5,6,7,8,9,10,11,12,13]:

(1) Variables related to the analysis of steel in the tundish, that is to say, the composition of the steel (solute). Three samples of the tundish per casting are sent to the laboratory for their analysis. Among these three samples, one of them is chosen as significant of the casting. The elements analyzed are:
  (a) Total manganese (Mn): The presence of sulfide is controlled by the addition of manganese. Manganese has a higher affinity for sulfur than iron so that instead of MnS, FeS is formed. FeS has a high melting point and good plastic properties. Manganese content should be about five times the sulfur concentration so that the reaction occurs. The end result, once removed causing gases, is a less porous casting, and therefore of higher quality.
  (b) Total sulfur (S): Its maximum limit is of about 0.04%. The sulfur along with iron gives place to iron sulfide, which with the austenite, results in a eutectic point with a low melting point and, therefore, it appears in the grain boundaries. When cast steel ingots are rolled in hot, this eutectic point is in liquid state, causing the shelling of the material. Although considered a detrimental element, their presence is positive for improved machinability in the machining processes. When the percentage of sulfur is high, it may cause pores in the welding process.
  (c) Total carbon (C): The term steel is commonly used to refer in metallurgical engineering to an iron alloy with a variable amount of carbon between 0.03% and 1.075% by weight of the alloy, depending on its applications and uses.
  (d) Total aluminum (Al): this alloying element is used in some high strength nitriding steels (with Cr-Al-Mo) at concentrations close to 1% and with percentages less than 0.008% as a deoxidizer in high alloy steels.
  (e) Total silicon (Si): this alloying element moderately increases the hardenability. Furthermore, it is used as a deoxidizing element. Additionally, it increases the resistance of low carbon steels.
  (f) Total phosphorus (P): this element is detrimental, either due to its dissolution in ferrite, which decreases the ductility, or due to formation of FeP. Its maximum limit is approximately 0.04%. Iron phosphide, along with the cementite and austenite, forms a ternary eutectic point called steadite, which is extremely fragile and has a relatively low melting point. Therefore, it appears in grain boundaries so that transmits brittleness to the material. Although it is considered a detrimental element in steels because it reduces their ductility and toughness, giving place to their brittle behavior, it is sometimes added to increase the tensile strength and improve machinability.
(2) Variables related to the cooling conditions of the slab:
  (a) Specific flow (Specific_Flow (m^3^·s^−1^)): The continuous casting machine is cooled. On the one hand, there is a primary cooling at the mold by using a water jacket (water casing) bolted to the plates. On the other hand, there is a secondary cooling in the rollers area through water showers. The value of the water flow injected to the rollers depends on casting parameters: type of steel, casting speed, temperature, *etc*. The specific flow is an index that determines the secondary cooling as a function of these parameters.
  (b) Average casting speed (m·s^−1^) (Ave_Speed): This variable is the average output speed of the slab from the casting machine. It influences on the solidification and cooling that it is necessary to apply.
  (c) Superheating in the tundish (Overtemperature) (°C): Steel begins to solidify when the temperature reaches a value called *liquidus temperature* and is different depending on its composition. For each of the samples taken in the tundish, three samples per casting, their actual temperature is measured and the liquidus temperature associated with each sample is calculated. The difference between the actual temperature and the liquidus temperature is known as overtemperature. This parameter is an important variable in the casting of steel since it measures how hot the steel is, if it is possible to cast it, and how fast. Thus, the colder is the slab, the faster it is casted, but if the steel is very cold, it is impossible to proceed with the casting process. Therefore, this parameter is of fundamental importance on the solidification and consequently on segregation.
  (d) Temperatures in Segment 8 and Segment 17 (°C) (Temp_Seg8 and Temp_Seg17): The rollers path of the casting machine is divided into groups of rollers called segments, which are numbered starting at the mold exit. In Segment 8 and Segment 17, there are pyrometers that measure the surface temperature of the slab as it exits the machine. Segment 8 is located on the curved zone of the machine and Segment 17 once the slab has been straightened. Their measurements may be regarded as indirect indicators of how the cooling process is performed.
  (e) Mold oscillation frequency (Freq_Oscillation): The mold is part of the continuous casting machine to give shape to the slab and where solidification begins. The mold rests on two eccentrics that impart an oscillatory motion to prevent the skin of the slab formed in the walls of the mold remains stuck to them. The frequency of this oscillation motion is fixed depending on the kind of steel casted. Its value must move the mold with a speed greater than the exit speed of slab.
  (f) Percentage of negative strip (Ratio_Strip): During the oscillatory motion of the mold, there is a time that the mold is moved downward faster than the line speed, which leads to an entrance effect of the slab into the mold. This represents a positive effect, decreasing the likelihood of formation of transverse cracks on the slab surface. The overall time of this effect is called percentage of negative strip.

All these variables described above have been selected as potential input variables of the model.

### 2.2. Segregation Evaluation

Traditional methods to evaluate central segregation in steel slabs from continuous castings consist either in etching with hydrochloric acid or in sulfur prints. The latter is the one obtained in this research work to obtain a segregation index acting as an objective variable in model development.

Sulfur prints, also known as Bauman impressions, are carried out according to the procedure contained in Reference [24]. It is a qualitative test that consists of the observation of the steel sulfur content. There are some factors such as the chemical composition of the steel, the state of the surface of the sample (specimen) and the characteristics of the photosensitive emulsion that can alter the results.

**Figure 2:**
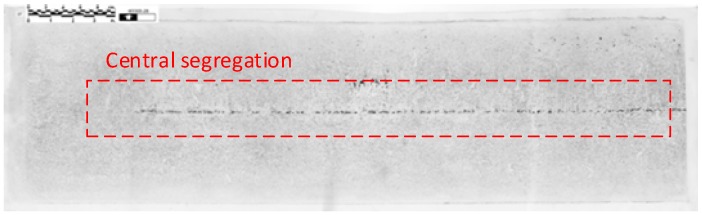
Example of a sulfur print.

A transversal section of the slab whose central segregation is wanted to be known is taken of length half of the slab width (the other half is symmetric). The sample is prepared by some mechanized action. On the other hand, the photographic paper is submerged in a sulfuric etching and is applied to the surface of the sample so etching takes place. Then, the paper is removed and washed with water. An example of a sulfur print can be seen in Figure 2.

Traditionally, sulfur prints were compared by an expert with some pattern images and a segregation index was obtained. However, this method is very subjective. Specifically, this paper uses a tool based on image processing. Indeed, this tool gives as output two indexes which measure the continuity and thickness of the segregated band. Furthermore, this tool could detect and measure cracks in the centerline. There are other methods for segregation like the one developed in Reference [25], which combines macroelectrolytic etching with image analysis [26] or the use of ultrasonic and computer aided analysis with a micro probe [27].

**Figure 3:**
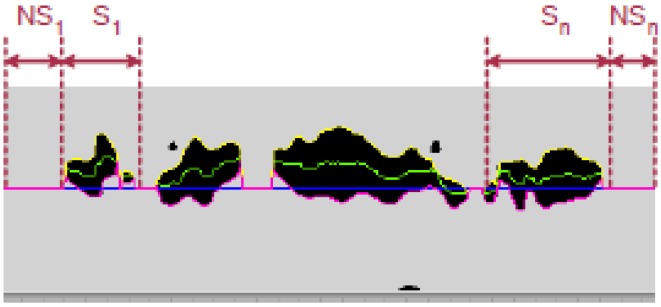
Upper, lower and mean segregation lines.

From the filtered and bitmapped images, this tool obtains different measures: the maximum and mean width of segregation line, the continuity of the segregation line, *etc*. In this way, the two main measures (continuity and width of the segregation centerline) are determined from the segregation’s mean line. This line is the median of the black pixels position of each column (see Figure 3).

Since big spots of segregation are more dangerous than small spots, the C factor takes into account this question computing the standard deviation $\sigma \left(S_{i}\right)$ and the mean size ${\bar{S}}_{i}$ of the continuous areas of segregation, and the standard deviation $\sigma \left(NS_{i}\right)$ and the mean size ${\bar{NS}}_{i}$ of areas without segregation, respectively. Its expression is as follows [4,5,6,7]:

(1) $$C\_Factor=\frac{\sum_{i=1}^{n}S_{i}}{\sum_{i=1}^{n}S_{i}+\sum_{i=1}^{n}NS_{i}}\times \left[\frac{\sigma \left(S_{i}\right)}{{\bar{S}}_{i}}+\frac{\sigma \left(NS_{i}\right)}{{\bar{NS}}_{i}}\right]$$

Finally, the width factor is calculated as the distance between the upper and lower line of the segregation spots and the Average Width as the mean of these widths (see Figure 3).

### 2.3. Segregation Models

Usually, segregation models are aimed to the comprehension of the mechanism implied in the phenomenon, predicting quantitatively their happening and severity and trying to improve casting procedures. Those models are very complex and require big computational efforts to simultaneously considerer all the processes during solidification. The first model of segregation was developed by Fleming *et al*. [28,29] during the 1960s. Based on this model, came out the work by Fleming (1974), Schneider and Beckermann (1995), and Gu and Beckermann (1999), among others [30,31,32]. More recent works are those by Ghosh (2001), Fujda (2005) and Liu *et al.* (2007) [33,34,35]. This research work presents a model of segregation based on the study of process data coming from the continuous casting machine using the MARS technique. Since there are no similar works reported in the literature, it has a very important innovative component. The objective of this research is to obtain a model of segregation based on process data from the continuous casting machine. This model will also be able to act as a predictor to infer the severity of segregation in a specific slab from the course of the casting process. The continuous casting of steel implies the online control of hundreds of process variables, so the modeling of centerline segregation requires of a previous stage of variable selection.

There are several modeling techniques used previously, such as MultiDimensional Scaling (MDS) [36,37], Sammon Mapping [38], Principal Component Analysis PCA [39], Feed Forward Neural Networks [40] and self-organizing maps [7]. In this paper, the selected procedure is the MARS technique [14,15,16,17,18,19,20,21,22,23], whose fundamentals are discussed below.

### 2.4. Method Multivariate Adaptive Regression Splines (MARS) Approach

Multivariate adaptive regression splines (MARS) is a multivariate nonparametric classification/regression technique introduced by Friedman [14,15,16,17,18,19,20,21,22,23]. Its main purpose is to predict the values of a continuous dependent variable, y(n×1), from a set of independent explanatory variables, x (n×p). The MARS model can be represented as [19,20,21]:

(2) y=f(x)+e

where *f* is a weighted sum of basis functions that depend on x and e is an error vector of dimension (n×1).

**Figure 4:**
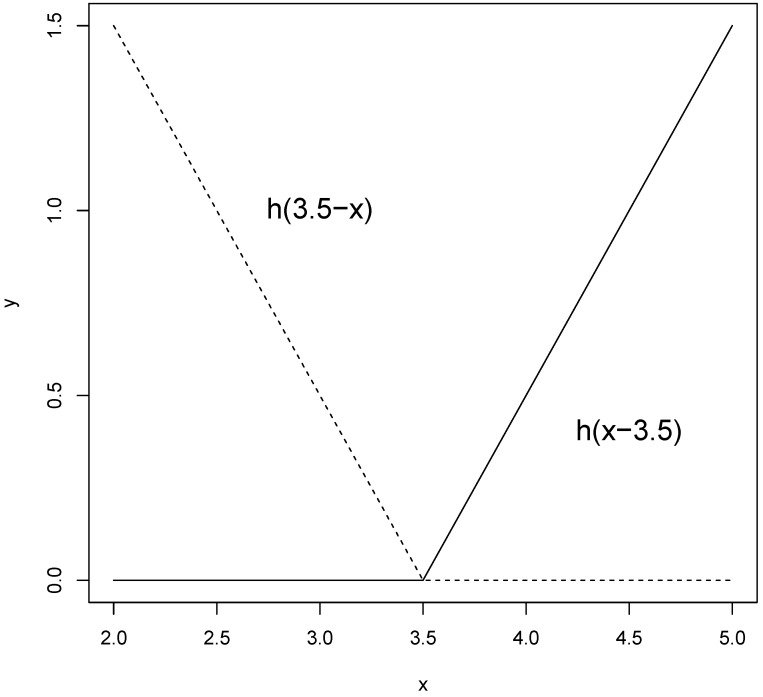
A graphical representation of a spline basis function. The left spline (x<t, −(x−t)) is shown as a dashed line and the right spline (x>t, +(x−t)) as a solid line.

MARS can be considered as a generalization of “classification and regression trees” (CART) [17,41,42] and is able to overcome some limitations of CART. MARS model does not require any *a priori* assumptions about the underlying functional relationship between dependent and independent variables. Instead, this relation is uncovered from a set of coefficients and piecewise polynomials of degree *q* (basis functions) that are entirely “driven” from the regression data (x,y). The MARS regression model is constructed by fitting basis functions to distinct intervals of the independent variables. Generally, piecewise polynomials, also called splines, have pieces smoothly connected together. In MARS terminology, the joining points of the polynomials are called knots, nodes or breakdown points. These will be denoted by the small letter *t*. For a spline of degree *q*, each segment is a polynomial function. MARS uses two-sided truncated power functions as spline basis functions, described by the following equations [14,15,16,17,18,19,20,21,22,23]:

(3) $${\left[-\left(x-t\right)\right]}_{+}^{q}=\{\begin{array}{cc} {\left(t-x\right)}^{q} & if\;\;x<t\\ 0 & otherwise \end{array}$$

(4) $${\left[+\left(x-t\right)\right]}_{+}^{q}=\{\begin{array}{cc} {\left(t-x\right)}^{q} & if\;\;x\geq t\\ 0 & otherwise \end{array}$$

where q (≥0) is the power to which the splines are raised and which determines the degree of smoothness of the resultant function estimate. When q=1, which is the case in this study, only simple linear splines are considered. A pair of splines for q=1 at the knot t=3.5 is presented in Figure 4.

The MARS model of a dependent variable y with *M* basis functions (terms) can be written as [14,15,16,17,18,19,20,21,22,23]:

(5) $$\hat{y}={\hat{f}}_{M}\left(x\right)=c_{0}+\sum_{m=1}^{M}c_{m}B_{m}\left(x\right)$$

where $\hat{y}$ is the dependent variable predicted by the MARS model, $c_{0}$ is a constant, $B_{m}\left(x\right)$ is the *m*-th basis function, which may be a single spline basis functions, and $c_{m}$ is the coefficient of the *m*-th basis functions.

Both the variables to be introduced into the model and the knot positions for each individual variable have to be optimized. For a data set *x* containing *n* objects and *p* explanatory variables, there are N=n×p pairs of spline basis functions, given by Equations (3) and (4), with knot locations $x_{ij}$ (i=1, 2,...,n;  j=1, 2,...,p).

A two-step procedure is followed to construct the final model. First, in order to select the consecutive pairs of basis functions of the model, a two-at-a-time forward stepwise procedure is implemented [21,41,42]. This forward stepwise selection of basis function leads to a very complex and overfitted model. Such a model, although it fits the data well, has poor predictive abilities for new objects. To improve the prediction, the redundant basis functions are removed one at a time using a backward stepwise procedure. To determine which basis functions should be included in the model, MARS utilizes the generalized cross-validation (*GCV*) [14,15,16,17,18,19,20,21,22,23]. In this way, the *GCV* is the mean squared residual error divided by a penalty dependent on the model complexity. The *GCV* criterion is defined in the following way [14,15,16,17,18,19,20,21,22,23]:

(6) $$GCV\left(M\right)=\frac{\frac{1}{n}\sum_{i=1}^{n}{\left(y_{i}-{\hat{f}}_{M}\left(x_{i}\right)\right)}^{2}}{{\left(1-C\left(M\right)/n\right)}^{2}}$$

where C(M) is a complexity penalty that increases with the number of basis functions in the model and which is defined as [14,15,16,17,18,19,20,21,22,23]:

(7) C(M)=(M+1)+d M

where *M* is the number of basis functions in Equation (4), and the parameter *d* is a penalty for each basis function included into the model. It can be also regarded as a smoothing parameter. Large values of *d* lead to fewer basis functions and therefore smoother function estimates. In our studies, the parameter *d* equals 2, and the maximum interaction level of the spline basis functions is restricted to 3 [14,15,16,17,18,19,20,21,22,23].

### 2.5. The Importance of Variables in the MARS Model

Once the MARS model is constructed, it is possible to evaluate the importance of the explanatory variables used to construct the basis functions. Establishing predictor importance is in general a complex problem, which, in general, requires the use of more than one criterion. In order to obtain reliable results, it is convenient the use of the *GCV* parameter explained before together with the parameters Nsubsets (criterion counts the number of model subsets in which each variable is included) and the residual sum of squares (*RSS*) [14,15,16,17,18,19,20,21,22,23].

## 3. Analysis of Results and Discussion

### 3.1. Results of The Model

The list of input variables taken into account in this research work is shown in Table 1. The total number of dependent variables (output variables) used to build the MARS models was two: *Continuity factor* (C_Factor) and the *Average Width* of the spots (Ave-Width) forming the centerline segregation. Indeed, we have built two different MARS models taking as dependent variables *C_Factor* and *Ave-Width*, respectively.

**Table 1:** Set of input variables used in this study with their mean and standard deviation.

Input variables	Name of the variable	Mean	Standard deviation
Total aluminum (measured as weight%)	Al	0.030	0.006
Total manganese (measured as weight%)	Mn	1.357	0.050
Total sulfur (measured as weight%)	S	0.009	0.002
Total carbon (measured as weight%)	C	0.173	0.014
Total phosphorus (measured as weight%)	P	0.016	0.004
Superheating (°C)	Overtemperature	24.545	8.940
Percentage of negative strip	Ratio_Strip	68.517	21.519
Specific flow (m^3^·s^−1^)	Specific_Flow	0.633	0.074
Average casting speed (m·s^−1^)	Ave_Speed	0.957	0.143
Mold oscillation frequency	Freq_Oscillation	2.043	0.688
Temperature in segment 8 (°C)	Temp_Seg8	816.472	265.506
Temperature in segment 17 (°C)	Temp_Seg17	771.911	246.454
Silicon (measured as weight%)	Si	0.201	0.048

In this research work, two second-order MARS models have been used, so that the basis functions of the model consist of linear and second-order splines and the maximum number of terms was not limited (no pruning). The results of the MARS models computed using all the available data observations are shown in Table 2 and Table 4. Table 2 and Table 3 show a list of 43 and 60 main basis functions for each of the two MARS models and their coefficients, respectively. Please note that h(x) = *x* if *x*>0 and h(x) = 0 if x≤0. Therefore, the MARS model is a form of non-parametric regression technique and can be seen as an extension of linear models that automatically models nonlinearities and interactions as a weighted sum of basis functions called *hinge functions* [14,15,16,17,18,19,20,21,22,23]. The predicted response for C factor (C_Factor) and average width (Ave–Width) is now a better fit to the original values since the MARS model has automatically produced a kink in the predicted dependent variable to take into account nonlinearities.

According to the results shown in Table 3, the most important variables for the prediction of the C factor (output variable) are as follows (in hierarchical order): Si, Temp_Seg8, S, Ratio_Strip, Mn, Temp_Seg17, Al, C, Overtemperature, P, Freq_Oscillation and Ave_Speed. Specific_Flow input variable is discarded by this model. Indeed, the most important variable is the silicon concentration (Si). This is due to that the silicon proceeds from the detachment of the refractory material during all the steel production steps.

**Table 2:** List of basis functions of the Method Multivariate Adaptive Regression Splines (MARS) model for the C factor (C_Factor) and their coefficients *c_i_*.

$B_{i}$	Definition	$c_{i}$
$B_{1}$	1	80.112
$B_{2}$	h (Ratio_Strip − 75.117)	−286.265
$B_{3}$	h (Ratio_Strip − 75.378)	471.796
$B_{4}$	h (Ave_Speed – 1.16)	6177.268
$B_{5}$	h (1.16 − Ave_Speed)	91.964
$B_{6}$	h (Temp_Seg8 − 870)	8.631
$B_{7}$	h (Temp_Seg8 − 889)	−20.522
$B_{8}$	h (889 – Temp_Seg8)	0.563
$B_{9}$	h (Temp_Seg8 − 906)	11.476
$B_{10}$	h (Al – 0.0247)	8358.107
$B_{11}$	h (Al – 0.0371)	−7741.410
$B_{12}$	h (Si – 0.2276) × h (889 – Temp_Seg8)	22.903
$B_{13}$	h (0.2276 – Si) × h (889 – Temp_Seg8)	−10.688
$B_{14}$	h (0.2483 − Si) × h (Temp_Seg8 − 870)	6.243
$B_{15}$	h (S – 0.0091) × h (Temp_Seg8 − 889)	433.489
$B_{16}$	h (0.0194 − P) × h (Temp_Seg8 – 906)	240.291
$B_{17}$	h (Freq_Oscillation – 2.43) × h (Ratio_Strip – 75.378)	697.928
$B_{18}$	h (75.378 – Ratio_Strip) × h (Temp_Seg8 − 953)	−30.322
$B_{19}$	h (75.378 – Ratio_Strip) × h (Temp_Seg8 – 938)	12.800
$B_{20}$	h (889 – Temp_Seg8) × h (Temp_Seg17 – 883)	0.433
$B_{21}$	h (881 – Temp_Seg8) × h (Al – 0.0247)	−35.436
$B_{22}$	h (Temp_Seg8 − 889) × h (0.0329 – Al)	537.071
$B_{23}$	h (Temp_Seg8 − 906) × h (Al – 0.0304)	−219.353
$B_{24}$	h (Temp_Seg8 – 906) × h (0.0304 – Al)	−961.240
$B_{25}$	h (C − 0.1863) × h (0.0091 − S) × h (Temp_Seg8 – 889)	−97083.453
$B_{26}$	h (C – 0.19) × h (75.378 – Ratio_Strip) × h (Temp_Seg8 – 953)	−16338.606
$B_{27}$	h (C – 0.1739) × h (Temp_Seg8 – 889) × h(Al – 0.0329)	114852.181
$B_{28}$	h (Mn – 1.3736) × h (0.0091 – S) × h (Temp_Seg8 – 889)	−16604.875
$B_{29}$	h (Mn – 1.3464) × h (889 – Temp_Seg8) × h (Temp_Seg17 – 883)	−11.470
$B_{30}$	h (1.3464 – Mn) × h (889 – Temp_Seg8) × h (Temp_Seg17 – 883)	38.383
$B_{31}$	h (0.2276 – Si) × h (P – 0.0166) × h (889 – Temp_Seg8)	503.269
$B_{32}$	h (Si – 0.2095) × h (75.378 – Ratio_Strip) × h (953 – Temp_Seg8)	−18.490
*B*_33_	h (0.2095 − Si) ×h (75.378 – Ratio_Strip) × h (953 – Temp_Seg8)	0.124
$B_{34}$	h (0.2483 − Si) × h (Ratio_Strip – 75.977) × h (Temp_Seg8 – 870)	−4789.996
$B_{35}$	h (0.2483 − Si) × h (Temp_Seg8 – 870) × h (Temp_Seg17 – 815)	−0.133
$B_{36}$	h (S – 0.0089) × h (Freq_Oscillation – 2.16) × h (899 – Temp_Seg8)	2206.549
$B_{37}$	h (S – 0.0089) × h (2.16 − Freq_Oscillation) × h (899 – Temp_Seg8)	59.436
$B_{38}$	h (0.0091 − S) × h (75.115 – Ratio_Strip) × h (Temp_Seg8 − 889)	20,180.563
$B_{39}$	h (S – 0.0091) × h (Overtemperature – 25) × h (Temp_Seg8 – 889)	−200.213
$B_{40}$	h (S – 0.0091) × h (25 − Overtemperature) × h (Temp_Seg8 – 889)	−36.885
$B_{41}$	h (0.015 – P) × h (75.378 – Ratio_Strip) × h (Temp_Seg8 – 870)	−1053.411
$B_{42}$	h (2.43 – Freq_Oscillation) × h (Ratio_Strip – 75.37) × h (Overtemperature – 29)	613.802
$B_{43}$	h (75.378 – Ratio_Strip) × h (953 – Temp_Seg8) × h (Al – 0.0383)	0.443
$B_{44}$	h (75.378 – Ratio_Strip) × h (953 – Temp_Seg8) × h (0.0383 – Al)	−0.306
$B_{45}$	h (Ratio_Strip – 75.378) × h (Temp_Seg8 − 870) × h (Al − 0.0314)	2183.857
$B_{46}$	h (Temp_Seg8 – 906) × h (Temp_Seg17 − 815) × h (0.0304 – Al)	3.265

**Table 3:** Evaluation of the importance of the variables that form the model for the C factor according to criteria Nsubsets, *GCV* and *RSS*.

Variable	Nsubsets	*GCV*	*RSS*
Si	45	100.0	100.0
Temp_Seg8	45	100.0	100.0
S	44	91.8	92.1
Ratio_Strip	44	91.8	92.1
Mn	43	86.5	86.8
Temp_Seg17	43	86.5	86.8
Al	42	81.0	81.4
C	33	58.5	57.3
Overtemperature	32	57.1	55.5
P	31	55.7	53.8
Freq_Oscillation	24	49.6	44.7
Ave_Speed	20	42.3	37.7

**Table 4:** List of basis functions of the MARS model for the average width (Ave_Width) and their coefficients *c_i_*.

$B_{i}$	Definition	$c_{i}$
$B_{1}$	1	0.2156
$B_{2}$	h (C − 0.1873)	−177.2487
$B_{3}$	h (0.1873 − C)	−29.2927
$B_{4}$	h (Si – 0.2483)	−45.3392
$B_{5}$	h (P–0.0174)	1102.0646
$B_{6}$	h (Ave_Speed – 1.16)	245.0236
$B_{7}$	h (1.16 − Ave_Speed)	8.2028
$B_{8}$	h (749 – Temp_Seg17)	−0.0064
$B_{9}$	h (Temp_Seg17 – 900)	−0.1186
**** $B_{10}$	h (Si – 0.02152) × h (Temp_Seg17 – 749)	0.3908
$B_{11}$	h (0.2152−Si) × h (Temp_Seg17 − 749)	1.0
$B_{12}$	h (S − 0.0074) × h (Temp_Seg17 − 749)	−4.3071
$B_{13}$	h (0.0146 − P) × h (Temp_Seg17 − 749)	−11.1187
**** $B_{14}$	h (P − 0.0166) × h (749 − Temp_Seg17)	1.8191
$B_{15}$	h (0.0166 − P) × h (749 − Temp_Seg17)	57.9514
$B_{16}$	h (Freq_Oscillation − 2.53) × h (Temp_Seg17 − 749)	0.1544
$B_{17}$	h (Ratio_Strip − 75.572) × h (Temp_Seg17 − 749)	0.0440
$B_{18}$	h (75.572 − Ratio_Strip) × h (Temp_Seg17 − 749)	0.0222
$B_{19}$	h (Temp_Seg8 − 921) × h (Temp_Seg17 − 749)	0.0015
$B_{20}$	h (921 − Temp_Seg8) × h (Temp_Seg17 − 749)	0.0002
$B_{21}$	h (Temp_Seg8 − 943) × h (Temp_Seg17 − 749)	−0.0017
$B_{22}$	h (Temp_Seg17 − 749) × h (Al − 0.0325)	3.2277
$B_{23}$	h (749 − Temp_Seg17) × h (0.0302 − Al)	0.7147
$B_{24}$	h (C − 0.1863) × h (0.0146 − P) × h (Temp_Seg17 − 749)	25,383.1351
$B_{25}$	h (0.1855 − C) × h (921 − Temp_Seg8) × h (Temp_Seg17 − 749)	−0.0074
$B_{26}$	h (1.4062 − Mn) × h (1.16 − Ave_Speed) × h (Temp_Seg17 − 749)	−0.7150
$B_{27}$	h (Mn − 1.3506) × h (921 − Temp_Seg8) × h (Temp_Seg17 − 749)	−0.0026
$B_{28}$	h (0.1979 − Si) × h (2.53 − Freq_Oscillation) × h (Temp_Seg17 − 749)	−3.1666
$B_{29}$	h (0.2152 − Si) × h (Freq_Oscillation − 2.45) × h (Temp_Seg17 − 749)	−6.1100
$B_{30}$	h (0.2152 − Si) × h (2.45 − Freq_Oscillation) × h (Temp_Seg17 − 749)	−2.4387
$B_{31}$	h (0.2152 − Si) × h (0.95 − Ave_Speed) × h (Temp_Seg17 − 749)	7.9399
$B_{32}$	h (0.1981 − Si) × h (921 − Temp_Seg8) × h (Temp_Seg17 − 749)	0.0111
$B_{33}$	h (0.1957 − Si) × h (Temp_Seg17 − 749) × h (Al − 0.0325)	132.0068
$B_{34}$	h (0.0074 − S) × h (P − 0.0127) × h (Temp_Seg17 − 749)	24,770.8361
$B_{35}$	h (S − 0.0074) × h (Ratio_Strip − 75.864) × h (Temp_Seg17 − 749)	118.2158
$B_{36}$	h (S − 0.0074) × h (Ratio_Strip − 75.977) × h (Temp_Seg17 − 749)	−190.8619
$B_{37}$	h (S − 0.0116) × h (921 − Temp_Seg8) × h (Temp_Seg17 − 749)	0.0704
$B_{38}$	h (P − 0.0156) × h (2.53 − Freq_Oscillation) × h (Temp_Seg17 − 749)	5.5200
$B_{39}$	h (0.0166 − P) × h (Freq_Oscillation − 1.62) × h (749 − Temp_Seg17)	−71.7430
$B_{40}$	h (0.0166 − P) × h (1.62 − Freq_Oscillation) × h (749 − Temp_Seg17)	−30.9687
$B_{41}$	h (P − 0.0146) × h (Ratio_Strip − 75.667) × h (Temp_Seg17 − 749)	−20.3425
$B_{42}$	h (P − 0.0146) × h (75.667 − Ratio_Strip) × h (Temp_Seg17 − 749)	−4.8084
$B_{43}$	h (P − 0.0166) × h (Ave_Speed − 0.88) × h (749 − Temp_Seg17)	−78.9370
$B_{44}$	h (0.0166 − P) × h (Ave_Speed − 1) × h (749 − Temp_Seg17)	−41.3467
*B*_45_	h (0.0166 − P) × h (1 − Ave_Speed) × h (749 − Temp_Seg17)	−280.7197
$B_{46}$	h (P − 0.0166) × h (Overtemperature−9) × h (749 − Temp_Seg17)	−0.0209
B_47_	h (P − 0.0146) × h (Temp_Seg8 − 879) × h (Temp_Seg17 − 749)	−0.1404
B_48_	h (P − 0.0146) × h (879 − Temp_Seg8) × h (Temp_Seg17 − 749)	−0.0658
B_49_	h (P − 0.0156) × h (Temp_Seg8 − 943) × h (Temp_Seg17 − 749)	0.2179
B_50_	h (0.0156 − P) × h (Temp_Seg8 − 943) × h (Temp_Seg17 − 749)	0.1261
B_51_	h (Freq_Oscillation − 2.04) × h (1.16 − Ave_Speed) × h (Temp_Seg17 − 749)	0.1828
B_52_	h (2.53 − Freq_Oscillation) × h (Ave_Speed − 1.09) × h (Temp_Seg17 − 749)	−3.6134
B_53_	h (2.53 − Freq_Oscillation) × h (804 − Temp_Seg8) × h (Temp_Seg17 − 749)	0.0013
B_54_	h (75.756 − Ratio_Strip) × h (921 − Temp_Seg8) × h (Temp_Seg17 − 749)	−0.0002
B_55_	h (Specific_Flow − 0.65) × h (Temp_Seg17 − 749) × h (0.0325 − Al)	−119.6246
B_56_	h (Overtemperature − 30) × h (Temp_Seg8 − 921) × h (Temp_Seg17 − 749)	−0.0005
B_57_	h (30 − Overtemperature) × h (Temp_Seg8 − 921) × h (Temp_Seg17 − 749)	−0.0001
B_58_	h (30 − Overtemperature) × h (Temp_Seg17 − 749) × h (0.0325 − Al)	0.1517
B_59_	h (Temp_Seg8 − 910) × h (Temp_Seg17 − 749) × h (Al − 0.0325)	−0.1202
B_60_	h (910 − Temp_Seg8) × h (Temp_Seg17 − 749) × h (Al − 0.0325)	−0.0317

Additionally, from the results shown in Table 5, it is possible to observe that the most important variables for the prediction of the average width of the spots (output variable) forming the centerline segregation are (in hierarchical order): S, P, Temp_Seg17, Ratio_Strip, Al, Temp_Seg8, Ave_Speed, Si, Overtemperature, Freq_Oscillation, Mn, C and finally, Specific_Flow. Indeed, the most important variable is the sulfur (S). In other words, a high percentage of sulfur in the composition of steel is detrimental to its properties, for example the pore formation during the welding process, *etc*.

**Table 5:** Evaluation of the importance of the variables that form the model for the *Average Width* of the spots according to criteria Nsubsets, generalized cross-validation (*GCV*) and residual sum of squares (*RSS*).

Variable	Nsubsets	*GCV*	*RSS*
S	30	100.0	100.0
P	29	55.3	56.0
Temp_Seg17	28	47.6	48.2
Ratio_Strip	28	47.6	48.2
Al	27	45.6	45.8
Temp_Seg8	21	29.6	29.1
Ave_Speed	20	26.6	26.2
Si	13	15.8	15.8
Overtemperature	10	12.7	12.7
Freq_Oscillation	44	78.3	70.5
Mn	43	77.3	69.0
C	39	73.0	62.9
Specific_Flow	8	31.6	24.6

**Figure 5:**
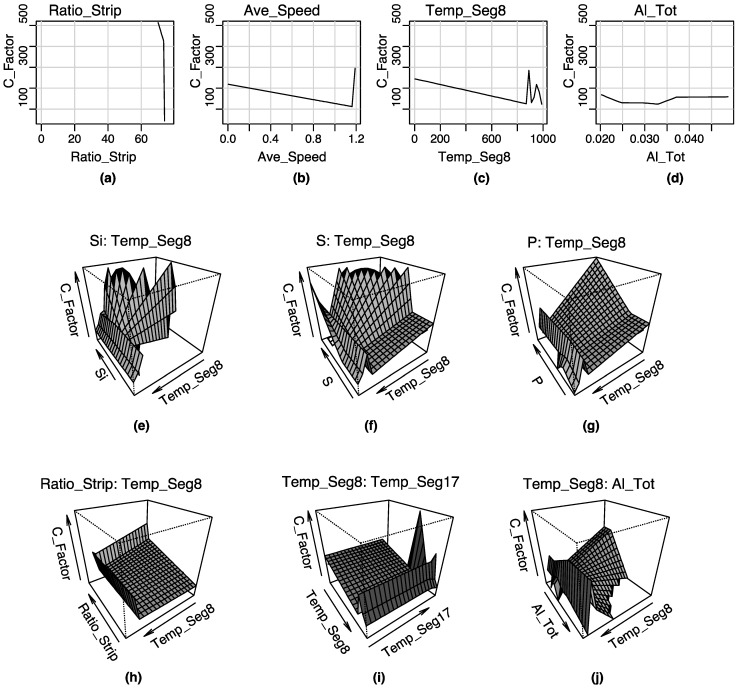
Graphical representation of the terms composing the MARS model for the C factor: (**a**) first order term of the predictor variable Ratio_Strip; (**b**) first order term of the predictor variable Ave_Speed; (**c**) first order term of the predictor variable Temp_Seg8; (**d**) first order term of the predictor variable Aluminum content; (**e**) second order term of the variables Si content and Temp_Seg8; (**f**) second order term of the variables Sulfur contents and Temp_Seg8; (**g**) second order term of the variables P content and Temp_Seg8; (**h**) second order term of the variables Ratio_Strip and Temp_Seg8 value; (**i**) second order term of the variables Temp_Seg8 and Temp_Seg17; (**j**) second order term of the variables Temp_Seg8 and Aluminum content.

**Figure 6:**
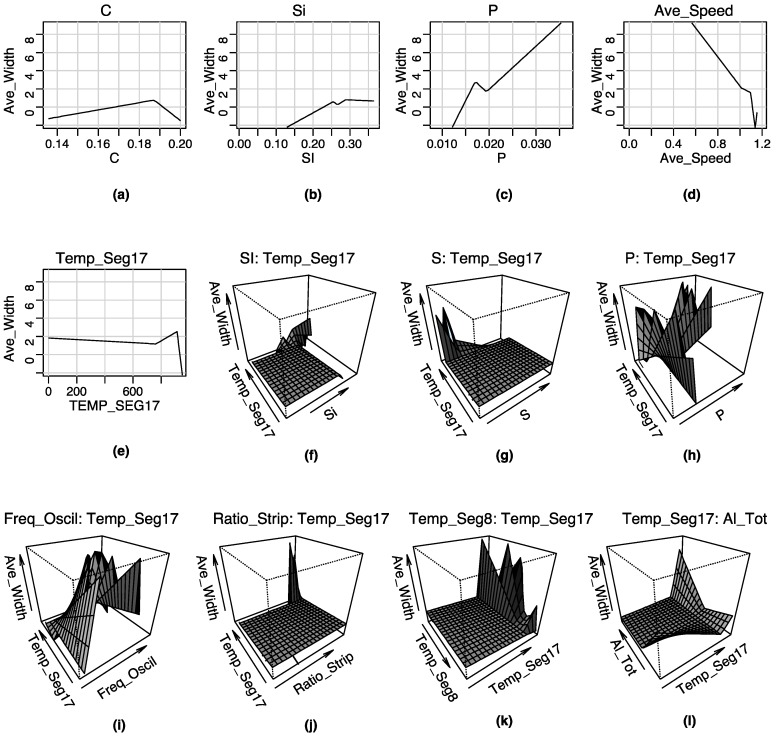
Graphical representation of the terms composing the MARS model for the *Average Width* of the spots forming the centerline segregation: (**a**) first order term of the predictor variable Carbon content; (**b**) first order term of the predictor variable Si; (**c**) first order term of the variable P; (**d**) first order term of the variable Average Speed; (**e**) first order term of the variable Temp_Seg17; (**f**) second order term of the variables Si and Temp_Seg17; (**g**) second order term of the variables S and Temp_Seg17; (**h**) second order term of the variables P and Temp_Seg17; (**i**) second order term of the variables Freq_Oscillation and Temp_Seg17; **(j**) second order term of the variables Ratio_Strip and Temp_Seg17; (**k**) second order term of the variables Temp_Seg8 and Temp_Seg17; (**l**) second order term of the variables Temp_Seg17 and Aluminum.

Furthermore, a graphical representation of the terms that constitute the two MARS models can be seen in Figure 5 and Figure 6, respectively.

### 3.2. The Goodness-Of-Fit for This Approach

It is important to select the model that best fits the experimental data. The following criterion was considered in this research: the coefficient of determination $R^{2}$ [43]. As it is well known, in statistics, the coefficient of determination is used in the context of statistical models whose main purpose is the prediction of future outcomes on the basis of other related information [17,41,42]. This ratio indicates the proportion of total variation in the dependent variables explained by the MARS model (C factor and average width of the spots in our case), that is to say, it provides a measure of how well future outcomes are likely to be predicted by the model. A dataset takes values $t_{i}$, each of which has an associated modeled value $y_{i}$. The former are called the observed values and the latter are often referred to as the predicted values. Variability in the dataset is measured through different sums of squares:

(1) $SS_{tot}=\sum_{i=1}^{n}{\left(t_{i}-\bar{t}\right)}^{2}$: the total sum of squares, proportional to the sample variance;
(2) $SS_{reg}=\sum_{i=1}^{n}{\left(y_{i}-\bar{t}\right)}^{2}$: the regression sum of squares, also called the explained sum of squares;
(3) $SS_{err}=\sum_{i=1}^{n}{\left(t_{i}-y_{i}\right)}^{2}$: the residual sum of squares.

In the previous sums, $\bar{t}$ is the mean of the *n* observed data:

(8) $$\bar{t}=\frac{1}{n}\sum_{i=1}^{n}t_{i}$$

(9) $$R^{2}\equiv 1-\frac{SS_{err}}{SS_{tot}}$$

A coefficient of determination value of 1.0 indicates that the regression curve fits the data perfectly. In this current research work, the two fitted MARS models for the *C factor* and *Average Width* of the spots have coefficients of determination equal to 0.93 and 0.95, respectively. These results indicate a very high goodness of fit for two MARS models analyzed.

Cross-validation is a model validation technique for assessing how the results of a statistical analysis will generalize to an independent dataset [44]. It is mainly used in datasets where the goal is prediction, and one wants to estimate how accurately a predictive model will work in practice. The aim of cross validation is to define a dataset to test the model in the training phase, in order to limit problems like overfitting, give an insight on how the model will generalize to an independent data set, *etc*. [45].

Therefore, in order to guarantee the ability prediction of the two built MARS models, the cross validation [44,45] was the standard technique used here for finding a suitable set of hyperparameters of the three MARS models built in this research work. In this sense, the data set is randomly divided into *l* disjoint subsets of equal size, and each subset is used once as a validation set, whereas the other l−1 subsets are put together to form a training set. In the simplest case, the average accuracy of the *l* validation sets is used as an estimator for the accuracy of the method. In this research work, 10-fold cross-validation was used, that is to say, to calculate the error criterion, the models were built using 90% of the sample and tested with the remaining 10%, thus simulating as closely as possibly the real conditions under which the model would be built in order to later fit it to new observation data unrelated to the construction of the models.

**Figure 7:**
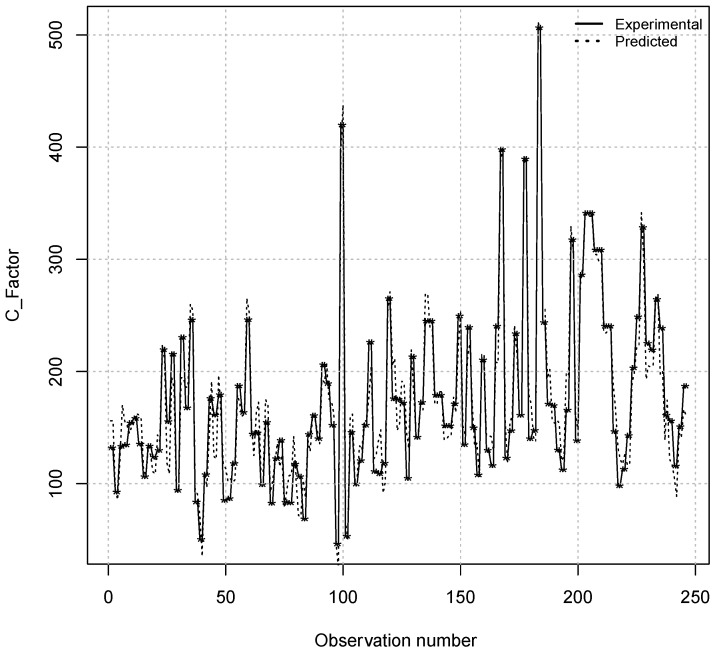
Comparison between the values of the *Continuity Factor* (C_Factor) observed experimentally and predicted by the model MARS from 245 actual observations.

**Figure 8:**
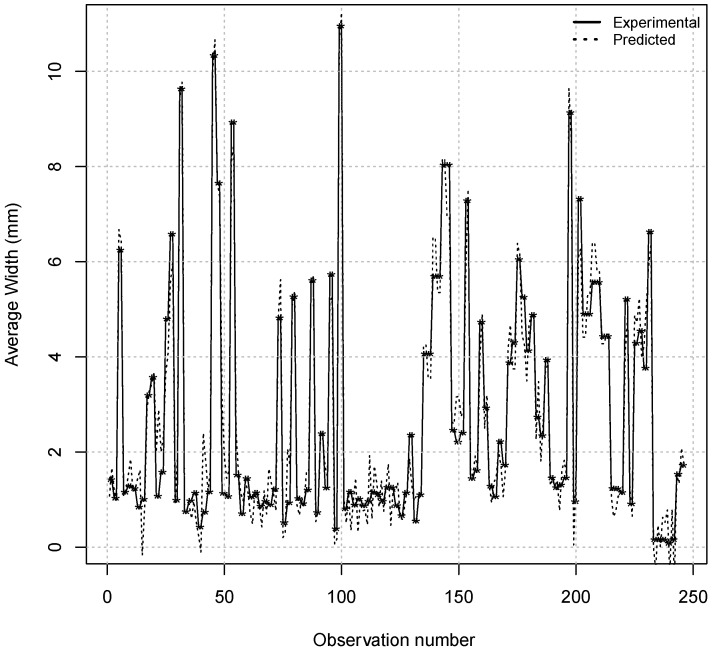
Comparison between the values of the *Average Width* of the spots forming the centerline of segregation observed experimentally and predicted by the model MARS from 245 actual observations.

Finally, this research work was able to estimate the values of the *Continuity Factor* from 245 experimental observations in agreement with the experimental actual values of *Continuity Factor* observed with success (see Figure 7). Similarly, Figure 8 shows a good agreement between the experimental concentrations of the average width of the spots forming the centerline segregation and their predicted values using the MARS models from 245 experimental observations, respectively. Indeed, coefficients of determination equal to 0.93 for Continuity Factor estimation and 0.95 for Average Width were obtained using this model, respectively.

Additionally, cross-validation is a model validation technique for assessing how the results of a statistical analysis will generalize to an independent dataset [45]. It is mainly used in datasets where the goal is prediction, and one wants to estimate how accurately a predictive model will work in practice. The aim of cross validation is to define a dataset to test the model in the training phase, in order to limit problems like overfitting, give an insight on how the model will generalize to an independent data set, *etc*.

In order to guarantee the prediction ability of this MARS model, an exhaustive cross-validation algorithm is used. Cross validation was the standard technique used in this research work in order to find the actual coefficient of determination of the model. The data set is randomly divided into *l* disjoint subsets of equal size, and each subset is used once as a validation set, whereas the other l−1 subsets are put together to form a training set. In the simplest case, the average accuracy of the *l* validation sets is used as an estimator for the accuracy of the method. In this way, 10-fold cross-validation was used [14,15,16,17,18,19,20,21,22,23,44,45].

Segregation is a very common and serious problem in steel production. The diagnostic techniques commonly used based on the traditional methods (such as to evaluate central segregation in steel slabs from continuous casting by etching with hydrochloric acid or with sulfur prints) are expensive from both the material and human standpoints. Consequently, the development of alternative diagnostic techniques is necessary. In this sense, the multivariate adaptive regression splines used in this work is a good choice to prevent segregation. The MARS is a nonlinear and non-parametric regression methodology and a flexible procedure that models complex relationships that are nearly additive or involve interactions with fewer variables. MARS exhibits the ability of modeling complex relationships among variables without strong model assumptions. Besides, MARS does not require a long training process and hence can save lots of modeling time when the data is particularly large. Therefore, the diagnostic model obtained using the MARS technique is a good methodology to predict the segregation and take measures in advance to tackle this problem. Indeed, this diagnostic technique requires low costs of implementation from both the material and human standpoints.

One of main goals in this research work was the study of the interactions among the input variables. Finally, the model developed in this research work was able to predict the segregation according to the actual database.

## 4. Conclusions

In this paper, a MARS model was used to make an estimation of segregation in steel labs continuously cast. The first conclusion obtained from the observation of the modeling results is the good agreement with the expert knowledge of the metallurgist about the phenomenon under study. It can be concluded that MARS can be a good machine learning technique to model this problem.

Based on the experimental and numerical results, the main findings of this research work can be summarized as follows:

Firstly, the hypothesis that segregation can be accurately modeled by using the MARS technique was confirmed. Two models were obtained. The first for the *Continuity Factor* and the second for the *Average Width* of the spots forming the centerline segregation.

Secondly, coefficients of determination equal to 0.93 for *Continuity Factor* estimation and 0.95 for *Average Width* were obtained when the MARS technique was applied to the experimental data set. The predicted results for the model have been proven to be consistent with the history of observed actual segregation.

Finally, one of the main findings of this study was to set the order of significance of the variables involved in the prediction of the output variables. On the one hand, the *Si* is the most influential variable in the *Continuity Factor* estimation. The second variable is the Temp_Seg8, the third variable is S, the fourth is Ratio_Strip, the fifth is Mn, the sixth is Temp_Seg17, the seventh is Al, the eighth is *C*, the ninth is Overtemperature, followed by P, Freq_oscillation and finally, the Ave_Speed. The Specific flow is unused for *Continuity Factor* estimation. On the other hand, Sulfur is the most influential variable in the *Average Width* estimation. The second variable is Phosphorous, the third variable is Temp_Seg17, the fourth is Ratio_Strip, the fifth is Al, the sixth is Temp_Seg8, the seventh is Average Speed, the eighth is Si, the ninth is Overtemperature, followed by Freq_oscillation, Mn, C and finally, the Specific_Flow.

In summary, this original and innovative methodology can be applied to another dataset with similar variables, but it is always necessary to take into account the specificities of each industrial metallurgical process. Additionally, the authors of this research work have confidence that the results obtained in this research will be useful to promote new future works in this line, developing other methodologies in predicting the segregation.
